# A phase I/II study of 4 monthly courses of high-dose cyclophosphamide and thiotepa for metastatic breast cancer patients

**DOI:** 10.1038/sj.bjc.6600631

**Published:** 2002-11-04

**Authors:** T Bachelot, F Gomez, P Biron, I Ray-Coquard, P Soler-Michel, I Philip, J P Guastalla, P Rebattu, A Dumortier, J P Droz, J Y Blay

**Affiliations:** Département de Cancérologie Médicale Centre Léon Bérard, 28, rue Laënnec, 69373 Lyon Cedex, France; Unité de Biostatistiques, Centre Léon Bérard, 28, rue Laënnec, 69373 Lyon Cedex, France

**Keywords:** filgrastim, haematopoietic stem cell transplantation, breast neoplasms, chemotherapy

## Abstract

This pilot phase I/II study intended to determine the maximum tolerated dose of cyclophosphamide and thiotepa administered on four consecutive courses with peripheral blood progenitor cell and granulocyte-colony stimulating factor support, as first-line therapy for hormone-refractory metastatic breast cancer patients. Twenty-eight patients were entered in the study. After two courses of epirubicin (120 mg m^−2^) and cyclophosphamide (2 g m^−2^) followed by granulocyte-colony stimulating factor injection and leukaphereses, patients received four cycles of cyclophosphamide and thiotepa. Each cycle was followed by peripheral blood progenitor cell and granulocyte-colony stimulating factor supports, then repeated every 28 to 35 days. Six escalating dose levels of cyclophosphamide and thiotepa were planned, beginning at cyclophosphamide 1.5 g m^−2^ and thiotepa 200 mg m^−2^. At least three patients were enrolled for each dose level. Eighteen patients completed the study. The maximum tolerated dose was 3000 mg m^−2^ cyclophosphamide and 400 mg m^−2^ thiotepa per course. Haematological toxicity was manageable on an outpatient basis and did not increase significantly with dose escalation. Dose-limiting toxicity was chemotherapy-induced immuno-suppression, which resulted in one toxic death and two life-threatening infections. Median times to treatment failure and survival were 11 and 26 months, respectively. Three patients were alive, free of disease 30 months after completion of the study. Such therapy allows for high-dose intensity and high cumulative doses on a short period of time with manageable toxicity.

*British Journal of Cancer* (2002) **87**, 1079–1085. doi:10.1038/sj.bjc.6600631 www.bjcancer.com

© 2002 Cancer Research UK

## 

The clinical course of metastatic breast cancer is variable, depending on individual responsiveness to treatment. Women with metastatic breast cancer are generally considered as incurable, with a median survival of about 2 years after documentation of metastases (Honig, 1996). Conventional chemotherapy regimens produce 40 to 80% objective response rates with median time to progression of less than 1 year (Clavel and Catimel, 1993; Nabholtz *et al*, 2001). Until now, high-dose chemotherapy (HDCT) regimens with autologous bone marrow or peripheral blood progenitor cell (PBPC) reinfusion have yielded high response rates, but demonstrated no survival improvement, in particular no obvious increase in the rate of long term survivors in currently available phase III trials (Stadtmauer *et al*, 2000; Antman, 2001). Hryniuk and Bush (1984) have suggested that chemotherapy regimens should be designed to maximise dose intensity rather than peak dose. This approach is further supported by the hypothesis of gompertzian growth of tumour cells *in-vivo*, which implies that multiple cycles of HDCT might be more effective than a single intensification course (Norton, 1988).

We planned the ERASM2A protocol as a phase I/II trial with the aim of developing an effective strategy allowing quadruple intensification with cyclophosphamide (CPA) and thiotepa (TTP) on an outpatient basis. These particular alkylating agents were chosen because (i) they are both highly potent against breast cancer, (ii) they lack major extra-haematological toxicity and (iii) they exhibit steep dose-response curves (Teicher *et al*, 1988; Eder *et al*, 1988).

## PATIENTS AND METHODS

### Patients

Patients who entered the study were required to have histologically proven metastatic adenocarcinoma of the breast with disease that was measurable or assessable (i.e. disease localisation not bi-dimensionally measurable but that could be monitored for complete response to treatment or progression). Patients should not have received chemotherapy for metastatic disease and their disease had to be hormone-refractory, i.e. receptor-negative for both oestrogen and progesterone, or progressive after hormonal therapy. Other requirements included: (1) age <61 years; (2) Eastern Cooperative Oncology Group (ECOG) performance status 0 or 1; (3) no adjuvant chemotherapy within 3 months of the diagnosis of metastatic disease; (4) normal left ventricular ejection fraction; (5) normal hepatic, renal and haematopoietic functions; (6) no brain metastasis; (7) no previous irradiation to the pelvis; and (8) no history of other malignancy. The protocol was approved by the *Comit*é *Consultatif de Protection des Personnes soumises* à *la Recherche Biom*é*dicale* of Lyon. Informed consent was obtained from all patients prior to entry in the study.

### Treatment plan

**Figure 1:**
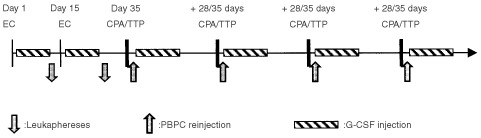
Treatment plan: Leukaphereses were performed after the first two courses of Epirubicin/Cyclophosphamide (EC). The four intensification courses with Cyclophosphamide and Thiotepa (CPA/TTP) were given every 28 to 35 days with PBPC and G-CSF support.

The study design is depicted in Figure 1

. The treatment schedule is enumerated from the first day of the first chemotherapy cycle. The first two cycles of chemotherapy consisted of epirubicin (EPI) 120 mg m^−2^ and CPA 2 g m^−2^ (EC regimen) given over 1 h at day 1 and day 15. G-CSF (filgrastim, 5 μg kg day^−1^) was administered daily by subcutaneous injection from day 5 to day 14, then from day 19 to day 28. Leukaphereses were performed on days 13, 14, 27 and 28. If necessary, subsequent leukaphereses were performed on days 29 and 30. A minimum of 10×10^6^ CD34+ cell kg^−1^ had to be harvested. This was the quantity required for five reinjections (four planned and one back-up). No treatment delays were allowed for the second EC regimen. When neutrophil count was below 1×10^9^ l^−1^ and/or platelet count was below 75×10^9^ l^−1^ at day 14, the second EC was administered with 90 mg m^−2^ EPI and 1.5 g m^−2^ CPA.

Four courses of high-dose CPA and TTP (CPA/TTP regimen) with G-CSF and PBMC support were administered every 4 to 5 weeks, starting at day 35. CPA and TTP were administered as a 48-h continuous infusion on days 35 and 36. Semi-saline hyperhydration (3 l m^2^ d^−1^) and mesna 1 g m^2^ day^−1^ was started at day 35, then carried on to day 37. PBPC (2×10^6^ CD34+ kg^−1^) were reinfused on day 39 and G-CSF (5 μg kg day^−1^) was started on day 40, then continued until the patient achieved a sustained neutrophil count over 0.5×10^9^ l^−1^ for 3 days. Patients were discharged from the hospital at day 39, then readmitted in case of febrile neutropenia or other severe toxicity. The four CPA/TTP cycles were repeated every 28 to 35 days. No treatment delays were allowed. If neutrophil count was below 0.5×10^9^ l^−1^ and/or platelet count was below 50×10^9^ l^−1^ at a maximum of 35 days from previous cycle, the patient was withdrawn from the study for haematological toxicity.

### Dose escalation

The starting doses of CPA and TTP were 1.5 g m^−2^ and 200 mg m^−2^, respectively. Six escalating dose levels were planned (Table 1

**Table 1:**
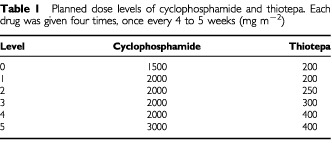
Planned dose levels of cyclophosphamide and thiotepa. Each drug was given four times, once every 4 to 5 weeks (mg m^−2^)

). Doses were assigned at registration and no dose-escalation was allowed in individual patients. Three patients were treated at each dose level. Before escalating to the next dose level, all three patients had to have completed the full treatment and then been observed for acute toxicity for at least 2 weeks. If any of the three patients developed a dose-limiting toxicity (DLT), one more patient was to be entered at the same dose level. If a second patient developed DLT, a fifth patient was to be entered at the same dose level. Finally, if a third patient at a given dose developed DLT, this dose level was defined as the maximum tolerated dose (MTD).

### Definition of dose-limiting toxicity

Toxicity was graded according to the National Cancer Institute Common Toxicity Criteria (NCI-CTC). DLT was defined as follows: Neutrophil count below 0.5×10^9^ l^−1^ and/or platelet count below 50×10^9^ l^−1^ at the theoretical starting day of a new CPA/TTP cycle (max. 35 days from previous cycle), or any grade 3 or 4 life-threatening non-haematological toxicity.

### Patients and treatment evaluation

Pre-study evaluation included medical history and physical examination, tumour measurement, CT-scans of head, chest and abdomen, bone scan, left ventricular ejection fraction, complete blood count, serum biochemistry plus liver function tests, tumour markers, bone marrow aspiration and biopsy.

Patients were monitored for toxicity at least weekly throughout treatment. Complete blood count was performed three times a week and serum biochemistry plus liver function tests twice a week. Assessment of tumour response was performed at the time of the first CPA/TTP cycle, then repeated 8 weeks after the fourth CPA/TTP cycle in the absence of clinical or biological evidence of early progression. Tumour response was assessed according to the WHO criteria.

### Statistical analysis

Statistical analyses were performed using Fisher's exact test, Mann–Whitney U-test or the log rank test when appropriate. Time to treatment failure (TTF) was measured from the date of inclusion to the time of progression or death or last follow-up, whichever came first, and assessed according to the Kaplan–Meier method. Statistical analysis was performed using SPSS 9.0 software (SPSS, Inc, Chicago, IL, USA).

Dose intensity (DI) was the total dose administered per square metre divided by the number of weeks of treatment (Hryniuk and Bush, 1984). For EPI, treatment duration was defined as the time from the first EC to the first CPA or to 28 days after the last chemotherapy cycle. For TTP, treatment duration was from the first day of the first CPA/TTP to 28 days after the last chemotherapy cycle actually received. For CPA, treatment duration was from the first EC to 28 days after the last chemotherapy cycle actually received. DI calculations for each treatment course only take into account the patients still receiving chemotherapy at that time.

## RESULTS

### Patient characteristics

Twenty-eight patients were enrolled into the study from January 6, 1995 to November 2, 1999 (Table 2

**Table 2:**
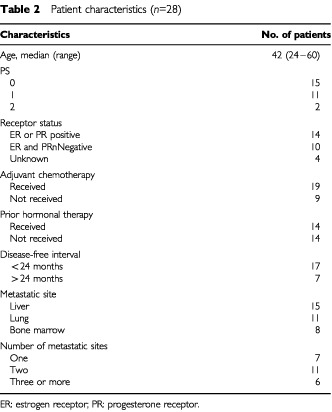
Patient characteristics (*n*=28)

). Median age was 42 years, with seven patients under 35 years. Most patients had visceral localisation and 24 had measurable lesions. The median disease-free interval was 1 year. Five patients were diagnosed with metastatic disease at presentation. Ten patients were receptor-negative for both oestrogen and progesterone. Of the 18 others, 14 had progressive disease after one, two or three lines of hormonal therapy, and four had rapidly progressive involvement of the liver requiring chemotherapy. Finally, 19 patients (68%) had initially received anthracycline-based adjuvant therapy.

### Intermediate dose induction therapy and leukapheresis

**Figure 2:**
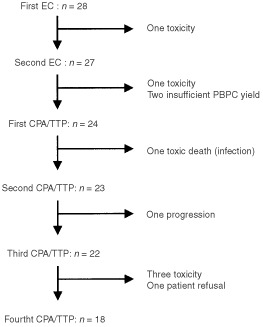
Flow chart of the treatment course for the 28 patients.

Twenty-seven patients received the two planned EC cycles (Figure 2

). One developed a *staphylococcus* infection on a hip implant after the first EC cycle and was withdrawn from the study. The EC regimen with G-CSF support was generally well tolerated, with no grade 3 non-haematological toxicity except from vomiting (three patients) and stomatitis (two patients). Haematological toxicity was substantial: 24 (86%) and 22 (81%) patients had to be readmitted for febrile neutropenia after the first and second EC cycles, respectively. Nevertheless, 25 (89%) patients received their second EC cycle on time (day 14 or 15) and at full doses. One patient needed a dose reduction and one was postponed for 12 days for persistent thrombopenia. Twenty-six patients required red blood cell transfusion and 15 required platelet transfusion. Leukaphereses were performed after the first and second EC. The median number of leukaphereses per patients was five (range 2–8). The median total CD34+ cells collected per patient was 18×10^6^ kg^−1^ (range 1.4–66.9).

### Intensification chemotherapy

Of the 27 patients who completed the induction treatment, two were withdrawn because of insufficient PBPC yield after six leukaphereses and one because of excessive haematological toxicity after the EC cycles with a persistent grade 3 thrombopenia at day 50 (Figure 2).

Twenty-four patients received at least one CPA/TTP cycle and a total of 87 courses were administered to this population. Eighteen patients (64% of the accrued population) received the four planned cycles. The dose of CPA and TTP were escalated through six dose levels from 1500 to 3000 mg m^−2^ of CPA and 200 to 400 mg m^−2^ of TTP (Table 1). One patient discontinued treatment after two cycles due to disease progression, two patients were withdrawn from the study for persistent thrombopenia after the third cycle, one patient died of toxicity after the first cycle, one was withdrawn after the third cycle for toxicity, and one refused therapy after the third cycle (Figure 2). Treatment timing was respected for most patients. The first CPA/TTP was administered before day 36 for all but nine patients for whom it was postponed for a median of 2 days (range 1–12 days). The second, third and fourth CPA/TTP cycles were given on time to all patients but one who received his fourth cycle on day 41 (median time from the previous cycle: 28 days; range 27–41 days). All patients were evaluable for toxicity.

### Dose intensity

The median time from the first EC cycle to the end of therapy (defined as four weeks after the last chemotherapy cycle actually received) for the 28 patients was 21 weeks (range 4–24 weeks). It was 22 weeks for the 18 patients who completed the study (range 21–24 weeks). Median total doses and dose intensity for the whole population are given in Table 3

**Table 3:**
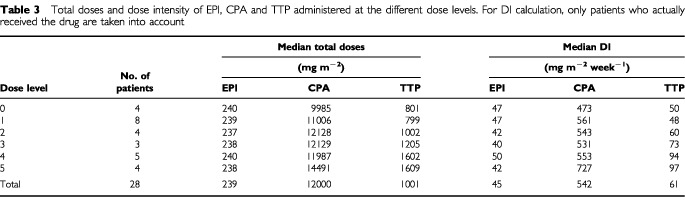
Total doses and dose intensity of EPI, CPA and TTP administered at the different dose levels. For DI calculation, only patients who actually received the drug are taken into account

.

### Haematological toxicity

Haematological toxicity was manageable on an outpatient basis. All cycles were followed by grade 4 neutropenia and most patients had to be readmitted for febrile neutropenia (defined by grade 2/3 fever and grade 4 neutropenia). Nevertheless, grade 4 neutropenia was of short duration, with a median of 4 days (range 1–8 days); patients were generally hospitalised for short periods, and hospitalisation length did not increase significantly with the number of cycles for one given patient, nor with dose escalation for the population as a whole (Table 4

**Table 4:**
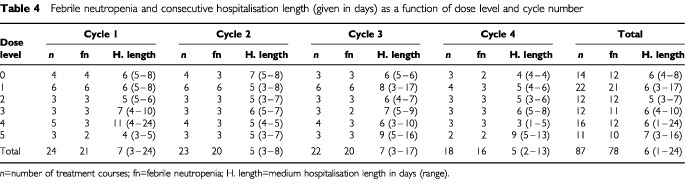
Febrile neutropenia and consecutive hospitalisation length (given in days) as a function of dose level and cycle number

).

Similarly, grade 4 thrombocytopenia occurred in 78 of the 87 cycles (90%), regardless of the dose level or the number of courses received. Platelet transfusion was given after 75 cycles (86%). The median number of platelet units transfused per patients at each cycle was 1 (range 0–4). Grade 3/4 anaemia requiring transfusion occurred after 78 cycles (90%). All patients received red cell transfusion with a median of 2 units per patients and per cycle (range 0–6). Finally, only two patients, at level 1, presented a haematological DLT, i.e. a persistent grade 4 thrombopenia at day 35 of a CPA/TTP cycle. Surprisingly, no other patient presented haematological DLT after dose escalation to levels 2 to 5.

### Non-haematological toxicity

Grade 3 and 4 non-haematological toxicities at each dose level are presented in Table 5

**Table 5:**
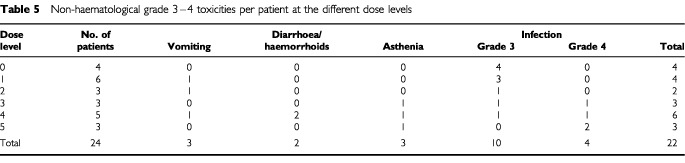
Non-haematological grade 3–4 toxicities per patient at the different dose levels

. Most non-haematological toxicities were expected and manageable. Alopecia (grade 2) was universal; one patient (level 4) experienced persistent grade 1 alopecia 2 years after completing treatment though she had received no other chemotherapy. Grade 2 vomiting occurred in 12 out of 24 patients, while grade 3 vomiting occurred in three; grade 2 nausea was observed in seven patients; grade 2 and 3 diarrhoea was reported in three and one patient, respectively. Constipation was rare (grade 2 in four patients) but haemorrhoids were frequent and sometimes severe (grade 2 in 6 patients and grade 3 in one patient, respectively). Most patients experienced mild stomatitis (grade 2 in 15 patients). Asthenia was significant with eighteen patients (75%) reporting grade 2 and two patients (8%) reporting grade 3 asthenia. One patient at level 5 refused the fourth CPA/TTP as a consequence of grade 3 asthenia, diarrhoea and vomiting. One patient experienced a transient increase in creatinine level (grade 2); no cardiac or neurological toxicity was reported. The major non-haematological toxicity was infection, with a total of 10 patients (42%) experiencing at least one episode of grade 3 infection with documented bacteriology. Four patients (17%) experienced grade 4 infection: One patient suffered from a septic shock following a *staphylococcus* septicaemia while in neutropenia after the second CPA/TTP at level 3. This episode lasted only 4 days and resolved without any sequel; One patient at level 4 died after the first CPA/TTP cycle from overwhelming sepsis caused by an antibiotic-resistant *enterococcus* while on intensive care for *pneumocystis* pneumonia; Two patients at level 5 developed a life-threatening lung infection after the third and fourth courses of CPA/TTP, respectively (one caused by *aspergillus* and one caused by *pneumocystis carinii*). As a consequence, we considered the MTD to be reached and the study was stopped at level 5.

### Initial lymphopenia and toxicity

Lymphopenia prior to the initiation of treatment is an independent predictive factor for chemotherapy-induced toxicity (Blay *et al*, 1998; Ray-Coquard *et al*, 1999). In this population, the median lymphocyte count before treatment was 1.25 G l^−1^ for the 18 patients who completed the treatment plan and only 0.8 for the 10 patients who did not (Mann–Whitney U-test, *P*=0.021). Among the six patients whose day 1 lymphocyte count was below 0.7×10^9^ l^−1^, only two completed the treatment. One died after the first CPA/TTP (toxicity); one was progressive after the second CPA/TTP; and two were withdrawn because of insufficient PBPC yield after six leukaphereses.

### Response and survival

At the time of the first CPA/TTP, 13 patients (46%) were considered by the investigator to be in partial response and 1 (4%) in complete response. Seven patients had stable disease, four were not evaluable for response but were not progressive, and three were not evaluated. Two months after completion of the study, six patients with stable disease after induction therapy were in partial response, one patient in partial response after induction therapy was in complete response, and three patients in partial response after induction therapy were progressive. At that time, the intention-to-treat overall response rate was 17 out of 28 (60%). Of the 24 evaluable patients, two had complete responses, 15 had partial responses, two had stable diseases and five had progressive diseases. Of the four patients with no measurable disease, two had progressive disease, one was not evaluable for response but was not progressive, and one was not evaluated (toxic death). The response rate appeared to be slightly higher in patients treated at dose levels 3–5, with an overall response rate of 75%, as compared to 50% for the 16 patients treated at dose levels 0–2 (Fisher's exact test, *P*=0.25).

**Figure 3:**
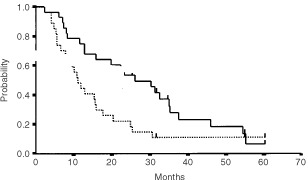
Time to treatment failure (dotted line) and overall (solid line) survival of the 28 patients from the first day of chemotherapy.

At the time of analysis, the median follow up was 26 months. The median TTF for the 28 patients was 11 months (95% confidence interval [CI]: 7–15; range 2–61+, Figure 3

). The median TTF of the 18 patients who completed the 4 cycles of CPA/TTP was 12 months (95% CI: 10–14; range 5–61+). The median overall survival was 26 months (95% CI: 15–37; range 2–61+, Figure 3). One patient had died of toxicity, 23 patients had died of disease progression, two were alive with progressive disease and three were alive with no evidence of progressive disease 31, 55 and 61 months after the first EC cycle. Of those three long survivors, one was in complete response, one in partial response and one in stable disease after the first 2 EC courses. Two were in complete response and one in partial response after completion of the study.

Finally, the median survival of the six patients whose lymphocyte count at day 1 was below 0.7×10^9^ l^−1^ was only 8 months, as compared to 34 months for the 18 other patients (log rank test, *P*<0.001).

## DISCUSSION

Only few phase I studies have explored the maximum tolerated dose of multiple sequential courses of HDCT in breast carcinoma, as well as in other tumours. This study was designed to determine the MTD of four courses of the association of high-dose CPA and TTP given over 4 months with PBPC and G-CSF supports. Induction therapy consisted of two rapidly cycled courses of CPA (2 g m^−2^) and EPI (120 mg m^−2^) with G-CSF supports followed by haematopoietic stem cell collection through leukaphereses. At the maximum tolerated dose, this protocol delivered 727 mg m^−2^ week^−1^ of CPA and 97 mg m^−2^ week^−1^ of TTP over a 5 month period, which represents a 4–6-fold increase of the doses of CPA as compared to conventional chemotherapy (Clavel and Catimel, 1993; O'Dwyer *et al*, 1991). In addition, this mode of delivery of CPA and TTP over four cycles enables to deliver twice as much total dose as a single course of HDCT with bone marrow support (Kennedy *et al*, 1991).

The association of G-CSF and PBPC supports allows rapid haematological recovery after each cycle, making haematological toxicities easily manageable on an outpatient basis (Basser *et al*, 1995; Rodenhuis *et al*, 1996; Viens *et al*, 1999). Interestingly, haematological toxicity as evaluated by the duration of neutropenia, thrombopenia, incidence of transfusion and delay to haematopoietic reconstitution did not significantly increase between courses one and four for all patients in this study. Similarly, no differences were observed between dose levels. Indeed, the DLT of this program of four sequential HDCT courses was not related to myeloid haematopoietic reconstitution.

In contrast, the rate of opportunistic infections, due to chemotherapy-induced cumulative immunosuppression was found to be the DLT of this study. Indeed, three opportunistic infections (two *pneumocistis* pneumonia, one invasive lung *aspergillus* infection), were observed in the highest two dose levels tested (eight patients). While these opportunistic infections are frequent after allogenous bone marrow transplantation, they are infrequently encountered after autologous bone marrow transplantation for solid tumours, in particular using PBPC (Hartmann *et al*, 1997). High-dose alkylating agents are known to impair T-cell immunity, particularly of CD4+ cells (Mackall *et al*, 1994). The administration of multiple HDCT courses results in a prolonged period of profound lymphopenia, exposing the patient to a risk of opportunistic infection (Mackall *et al*, 1994; Greenberg and Riddell, 1999). Nevertheless, this treatment was associated with little life-threatening toxicity and only one treatment-related death.

Extra-haematological toxicities reported in this study of multiple cycles of high-dose chemotherapy courses were found to be different from the toxicity profile of single very high-dose treatments using the same drugs (Kennedy *et al*, 1991; Eder *et al*, 1990; Wolff *et al*, 1990; Vahdat *et al*, 1995). Severe mucositis and neurological toxicities where reported in single high dose treatment while in this study mucositis was frequent but manageable (grade 2 in 62% of the patients) and central nervous system toxicity was not observed in any patient. This is likely to be related to the specific toxicity of very high single doses of TTP (>700 mg m^−2^) that were not reached in this study. Alternatively, as no pharmacokinetics analysis has been made, we cannot rule out a metabolic interaction between CPA and TTP. It has been described that continuous infusion of TTP may inhibit the activation route of CPA (Huitema *et al*, 2000). This could account for the absence of typical high-dose CPA toxicities, such as haemorrhagic cystitis. Of note, treatment-related fatigue was observed in a significant proportion (83%) of patients. This side effect is also less frequently reported in studies of single HDCT.

Despite young age (median 42 years) and lack of prior therapy for metastatic disease, only 18 out of 28 patients (64%) were able to complete the whole treatment plan, whereas 10 were withdrawn during treatment, two of them due to insufficient PBPC collection and seven due to excessive toxicity. Several other groups have tested the association CPA/TTP in multiple-dose intensive chemotherapy with different protocols. Vahdat *et al* (1995) administered 2 courses of CPA (3 g m^−2^ course^−1^) and 2 courses of TTP (500–700 mg m^−2^ course^−1^) to 42 patients over a 2-month period. Most patients completed the study (90%), neurological toxicity prevented further escalation of TTP. Two published studies tested the CTC association (TTP, CPA, carboplatin,) on multiple courses with PBPC support. In the report from Shapiro *et al* (1997), four courses of caboplatin (200 mg m^−2^), CPA 1500 mg m^−2^ and TTP 125 mg m^−2^ were given every 21 to 42 days. Toxicity was moderate, with no toxic death, but, as in our series, only 67% of the patients could complete the treatment due to toxicity or disease progression. Rodenhuis *et al* (1996) reported the results of a much more aggressive protocol which consisted of carboplatin (1600 mg m^−2^), CPA (6000 mg m^−2^) and TTP (480 mg m^−2^) for three courses every 28 days. Toxicity was not acceptable after the second course. In a subsequent trial with a 33% dose reduction, eight breast cancer patients were treated with ‘reasonable’ toxicity. Those results in terms of doses and toxicities are consistent with our report. Taken together, they show that despite manageable haematological toxicity in an outpatient setting, multiple-dose intensive regimens would hardly be tolerated by most patients in general practice, particularly in the case of older patients (Antman, 2001).

The overall response rate (60%), median time to treatment failure (10 months) and median survival (26 months) achieved with this regimen is comparable to that of previous studies of high-dose therapy for metastatic disease. However, in these previous studies, patients were randomised only if they responded to conventional chemotherapy (Stadtmauer *et al*, 2000; Antman, 2001). In this series, three patients were alive, free of progressive disease 31, 55 and 61 months after treatment, respectively. All had cytologically confirmed metastasis. This treatment approach may therefore be curative in a small subset of patients.

In view of toxicity and long term survival rates, patient selection will be of major importance in future studies, both to prevent unacceptable toxicities and to discriminate the few patients who are most likely to have a long-lasting response to this therapy. It had been previously reported that the lymphocyte count was an independent prognostic parameter of the toxicity of chemotherapy, as well as a predictor of survival (Blay *et al*, 1998; Ray-Coquard *et al*, 1999; Ray-Coquard *et al*, 2001). The present results support these observations and further suggest that lymphocyte count could be a useful parameter to evaluate the feasibility and toxic risks of multiple courses of high-dose chemotherapy programs in clinical practice. Nevertheless, this series was too small to perform a complete statistical analysis that would take into account other classical parameters. Further studies are required to assess the importance of lymphopenia as a predictive factor for toxicity in this setting.

In conclusion, we have shown that a regimen associating four consecutive chemotherapy courses of high-dose CPA and TTP with G-CSF and PBPC supports allows the administration on a 4-month period of high dose-intensity and very high cumulative drug doses. Toxicity was important, with one toxic death and only 18 of 24 patients completing the study, but manageable on an outpatient basis. Response and survival are promising but few patients may actually have a substantial survival benefit from this treatment. Better patient selection procedures are essential before new randomised trials are initiated.
